# Removal of Cyanoacrylate Glue From the External Auditory Canal With Hydrogen Peroxide and Acetic Acid: A Case Report

**DOI:** 10.7759/cureus.57213

**Published:** 2024-03-29

**Authors:** Jatin Ahluwalia, Emily Drury, Michael Haupert

**Affiliations:** 1 Otolaryngology, Ascension St. John Hospital, Detroit, USA; 2 Pediatric Otolaryngology, Ascension St. John Hospital, Detroit, USA

**Keywords:** otology, foreign body, hydrogen peroxide, hearing loss, acetic acid, external auditory canal, superglue, cyanoacrylate

## Abstract

Ear canal foreign bodies are commonly encountered in the field of otolaryngology. This is especially common in the pediatric otolaryngology discipline. As a foreign body, cyanoacrylate glue (also called "super glue") can be difficult to remove and cause significant patient distress. Multiple solvents can be described as aiding in removing such foreign bodies. Here, a case is described in which hydrogen peroxide and acetic acid were used sequentially to remove cyanoacrylate glue from the external auditory canal. We describe a technique allowing en bloc removal of the cyanoacrylate glue. Thankfully, the patient's hearing returned to baseline post-operatively with minimal complications. Overall, cyanoacrylate glue can be a very difficult foreign body to remove from the ear canal. In this case, the sequential use of hydrogen peroxide and acetic acid to soak the glue was a safe and successful method to facilitate glue removal from the ear canal.

## Introduction

Foreign bodies getting stuck in the ear is a common problem encountered in the field of otolaryngology [1]. Removal and management often require the expertise of an otolaryngologist to minimize complications [2]. Cyanoacrylate glue, often referred to as "super glue", is a substance that can get stuck in the external ear canal (EAC) and cause a plethora of problems. These problems include pain, bleeding, hearing loss, damage to the tympanic membrane (TM), and patient anxiety [3]. These complications stem from cyanoacrylate's ability to cause local tissue toxicity and rapid tissue degradation as it releases several toxins [4]. Cyanoacrylate is an acrylic resin that polymerizes in alkaline substances. Once polymerized, cyanoacrylate hardens and becomes very rigid [5]. Because cyanoacrylate is a very strong adhesive that bonds inanimate objects to the skin, removal without damage to the EAC and TM poses a significant challenge [6]. Cyanoacrylate can be dissolved in acetone, methyl ethyl ketone, nitromethane, and methylene chloride. Acetone is a common solvent used to remove cyanoacrylate glue from skin in general [7]. However, acetone and other organic solvents are known to be ototoxic, specifically targeting the cochlear outer hair cells, and can lead to permanent sensorineural hearing loss [8]. Acetic acid is another substance that has been used as an alternative to acetone. However, there have been reports of ototoxicity secondary to acetic acid use as well [9]. Therefore, multiple agents may be preferred for removing cyanoacrylate-based glues from the EAC to minimize complications. This case report illustrates the subsequent sequential use of hydrogen peroxide and acetic acid to remove cyanoacrylate glue safely and easily from the EAC.

## Case presentation

**Figure 1:**
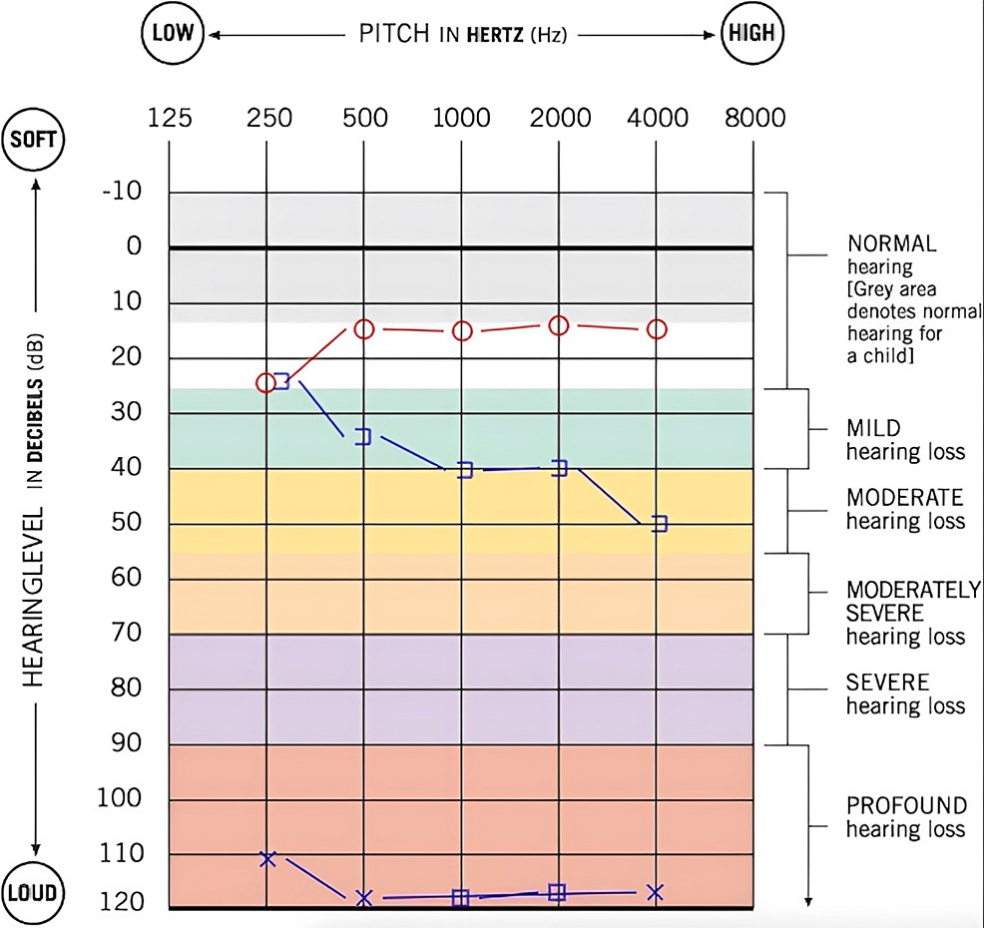
A pre-operative audiogram reveals profound mixed hearing loss in the left ear and normal hearing in the right ear Red circle: unmasked air conduction threshold in the right ear; Blue bracket: masked bone conduction threshold in left ear; Blue X: unmasked air conduction threshold in the left ear; Blue square: masked air conduction threshold in the left ear

A 15-year-old male with a past medical history of depression, attention-deficit/hyperactivity disorder (ADHD), and non-epileptic seizures presented to the pediatric otolaryngology clinic with the complaint of glue in the left ear canal and hearing loss. He reported that one week before the presentation in the clinic, he had an auditory hallucination in which he was told to put glue in his ear to ruin his hearing. He had already been following up with a psychiatrist and undergoing therapy for these auditory hallucinations. Upon discussion, it was revealed that he did insert cyanoacrylate glue in his left ear and has had persistent hearing loss in that ear since the incident. Before this, he reported normal hearing in both ears. He denied dizziness or vertiginous episodes. He had not attempted removal or application of substances into the ear before evaluation in the clinic. On exam, the left external auditory canal was completely occluded with hard, dried glue, partially extended laterally along the conchal bowl. The right external auditory canal was patent, and the tympanic membrane was intact and mobile. An audiogram was performed, and it showed profound mixed hearing loss in the left ear and hearing within normal limits for the right ear (Figure 1). The concerning findings on the audiogram and the potential for permanent hearing loss were discussed with the patient's mother. A plan was then made to board the patient for foreign body removal of the left external auditory canal in the operating room.

**Figure 2:**
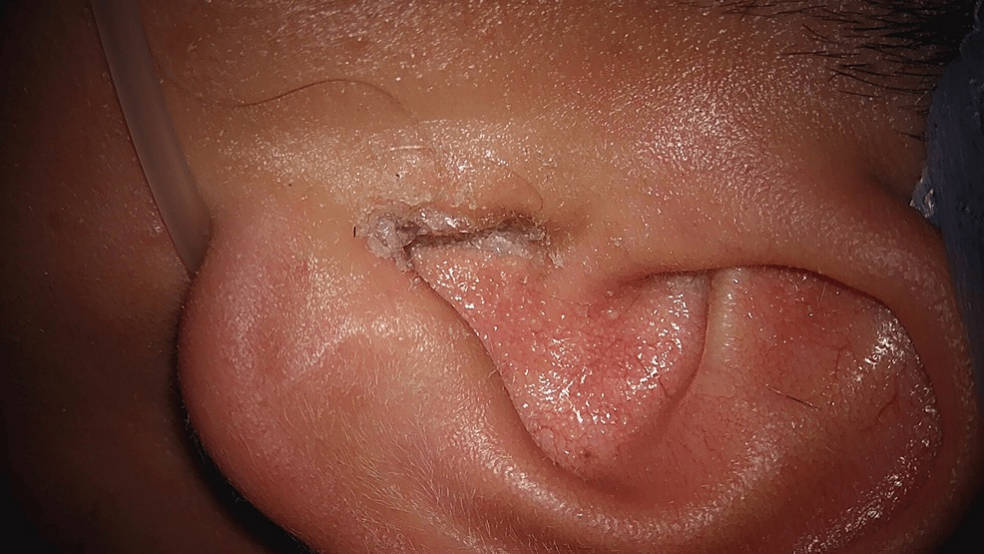
Cyanoacrylate glue completely occluding and extending laterally from the left external auditory canal

**Figure 3:**
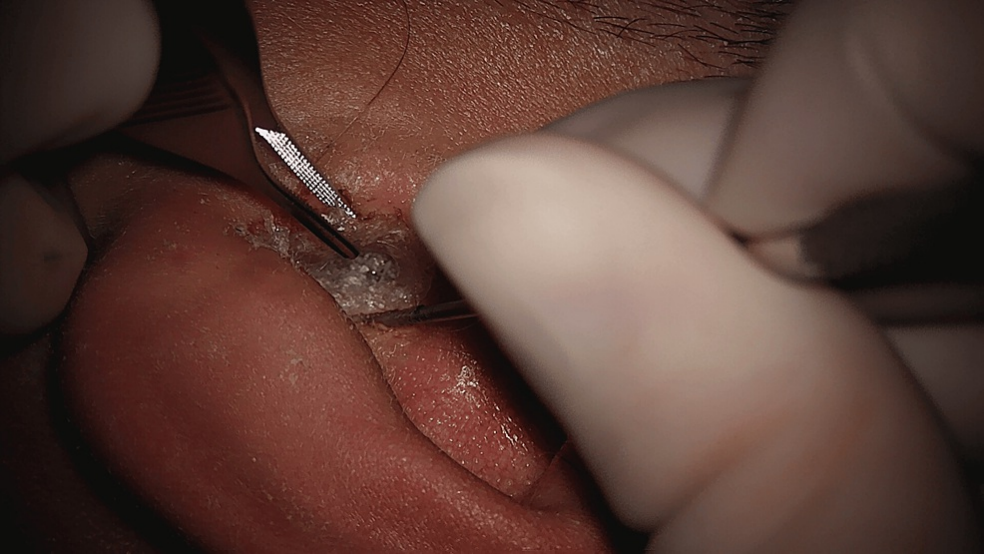
Probing around superglue to separate from its surrounding skin after soaking with hydrogen peroxide and acetic acid

**Figure 4:**
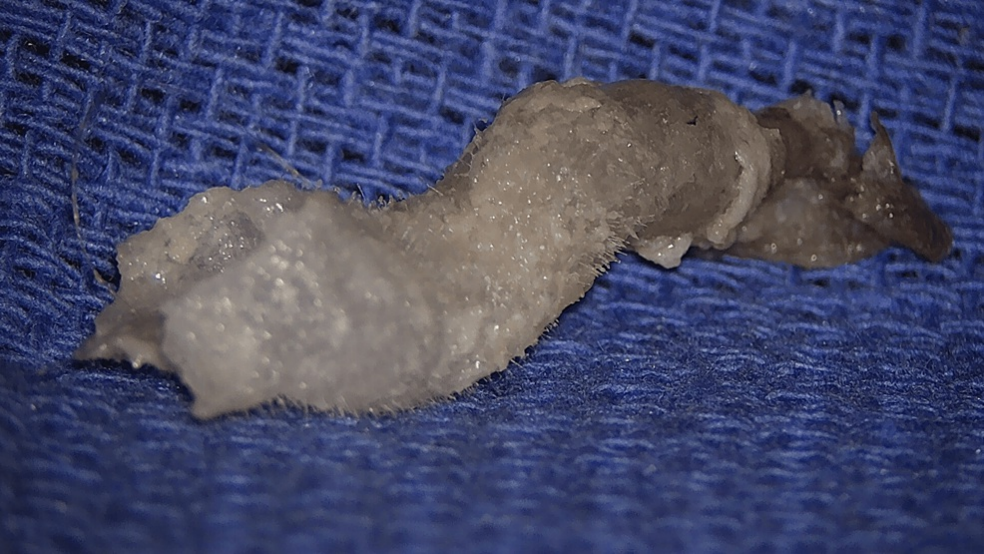
En bloc removal of cyanoacrylate glue from the left external auditory canal

The patient was boarded for the operating room five days later (12 days after the initial application of superglue). Before surgery, the patient was instructed to soak the left ear canal and glue it with 3% hydrogen peroxide for 30 minutes. After this initial soak was completed, the patient was taken into the operating room and induced with general anesthesia. Under visualization with a microscope, hydrogen peroxide was again generously applied to the glue and left external canal. The glue was noted to extend laterally out of the external auditory canal (Figure 2). Next, the glue was soaked with 4% acetic acid and probed to free it from the skin, which seemed to free it easily (Figure 3). This process of soaking with acetic acid and probing around the glue to free it from the skin was repeated until it was entirely removed in one piece (Figure 4). The glue appeared to have a layer of cerumen attached at the medial end that would have been adjacent to the tympanic membrane. The microscope was used to inspect the ear canal and tympanic membrane. The ear canal appeared erythematous and edematous, and the tympanic membrane was intact. All instrumentation was removed, and the patient was transported to the recovery room, tolerating the procedure well. Post-operatively, he was prescribed ciprofloxacin-dexamethasone otic drops for the left ear for 10 days.

**Figure 5:**
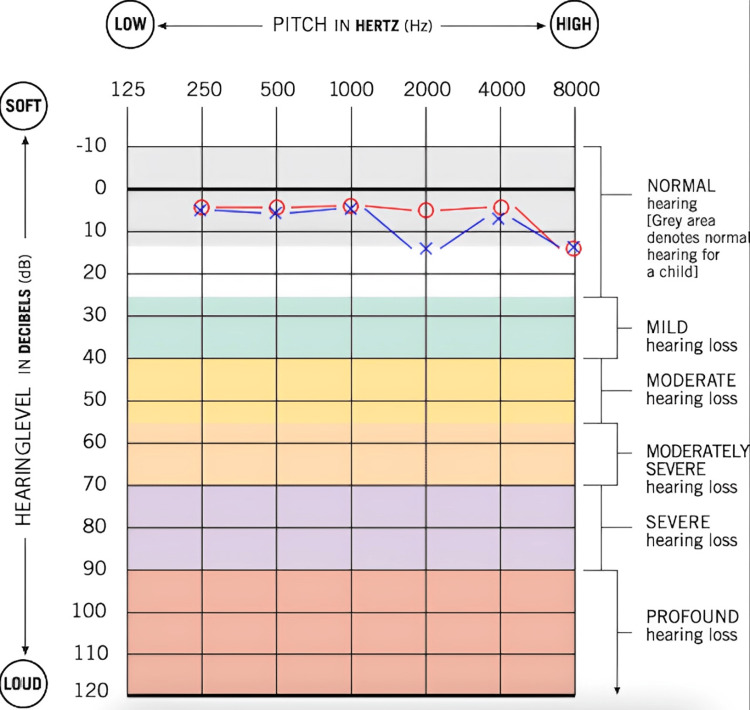
Post-operative audiogram revealing normal hearing bilaterally Red circle: unmasked air conduction threshold in the right ear; Blue X: unmasked air conduction threshold in the left ear

The patient was then evaluated three weeks post-operatively. The patient reported his hearing had returned to normal. He denied any otologic complaints. On exam, his left external auditory canal was patent, with no evidence of erythema, edema, or scarring. An audiogram was performed, showing hearing within normal limits bilaterally (Figure 5). Tympanogram and otoacoustic emission testing (OAE) were also obtained within normal limits. It appeared the patient had not suffered any permanent audiological damage from this incident. He was instructed to follow up as needed and avoid putting foreign bodies in the ears in the future.

## Discussion

Ethyl cyanoacrylate is commonly used in "super glue" products [10]. Since this compound can cause local toxicity and skin and tissue degradation, placing this material within the external auditory canal poses a difficult challenge. Immediate complications of this include direct damage to the skin and potentially underlying cartilage and bone of the external auditory canal [11]. This type of insult would increase the patient's risk of developing an acute otitis externa, which may lead to worsening patient morbidity and increased need for medications and follow-up. More concerning, however, is when a large amount of this material is placed within the external auditory canal, as was presented in this case report, the risk of developing direct damage to the tympanic membrane increases. Specifically, the risk of tympanic membrane perforation and total avulsion of the tympanic membrane put that patient at significant risk of developing both a conductive and sensorineural type hearing loss [12]. The presence of superglue alone within the EAC would be sufficient to yield conductive hearing loss, but this effect may be worsened should the glue accumulate within the middle ear space and affect the conduction of sound through the ossicular chain. Furthermore, should the superglue enter the middle ear space, there is the potential to damage the ossicles and potentially cause direct injury to the auditory hair cells within the cochlea [13]. Given these risks, an audiogram should be obtained at the time of the initial visit, if possible, to establish a baseline and to assess for possible mixed hearing loss.

In this case, the patient presented with a profound mixed hearing loss on the affected side, suggesting a component of inner ear toxicity and the possibility of irreversible hearing loss. Because of this, it was surprising to find the tympanic membrane completely intact without evidence of perforation. Additionally, post-operative audiometry was essentially within normal limits and symmetrical bilaterally. This was a very interesting finding given the mixed hearing loss present on a pre-operative audiogram. It is possible that this may have been due, in part, to variability in the patient's attentiveness during audiological testing.

Prompt surgical evaluation is generally recommended should there be a large amount of superglue present that fails to be removed with observation and conservative measures. Typically, desquamation of the epidermis does occur within 1-2 days should the superglue mass remain present; however, given the size and shape of the external auditory canal, this alone is typically insufficient for the removal of the glue without the aid of solvents [14]. While cyanoacrylate glues are soluble in acetone, methyl ethyl ketone, and nitromethane, just to name a few, these solutions do not come without risk. Acetone is most widely used; however, it does pose a risk of ototoxicity and should be used with caution in settings where the possibility of a tympanic membrane perforation has not been entirely excluded [15]. Hydrogen peroxide has also been used as an adjunct solvent to aid the removal of solidified superglue mass, but this, too, comes with the risk of local tissue irritation, given its corrosive properties [16]. There have been documented reports of the application of cyanoacrylate glues to the nasal and oral cavities as well. In these circumstances, the use of solvents is generally avoided, given the higher risk of local mucosal injury [17]. The presence of skin within the external auditory canal, therefore, allows for more judicious use of solvents, which should be taken advantage of. Overall, using multiple solvents may be advantageous when attempting the removal of cyanoacrylate-based compounds from the ear.

## Conclusions

Foreign bodies of the ear are a relatively common presentation in the field of otolaryngology. Cyanoacrylate glue can be a very difficult foreign body to remove from the ear canal due to its adhesive properties and local tissue effects. Administration of general anesthesia is often required for removal. Many of the traditional solvents used to aid the removal of cyanoacrylate glue, including acetone and acetic acid, possess ototoxic properties. Therefore, using multiple agents may be advantageous in assisting with removal. In this case, sequentially using hydrogen peroxide and acetic acid was a safe and successful method to facilitate en bloc removal of the glue from the ear canal.
